# Evaluating the iliotibial band as a biological internal brace in ACL repair surgery: a finite element analysis study

**DOI:** 10.3389/fbioe.2026.1913327

**Published:** 2026-09-30

**Authors:** Pooyan Vahidi Pashaki, Benjamin Noonan, Kalpana S. Katti, Dinesh R. Katti

**Affiliations:** 1 Department of Civil Construction and Environmental Engineering, North Dakota State University, Fargo, ND, United States; 2 Sanford Health, Fargo, ND, United States

**Keywords:** anterior cruciate ligament (ACL), biological internal brace, finite element analysis, IT band-ACL repair, surgery

## Abstract

**Introduction:**

The anterior cruciate ligament (ACL) is essential for knee stability but has limited regeneration ability due to low cell density and poor vascularization. Current ACL repair and augmentation approaches aim to preserve native tissue and provide early mechanical stability; however, challenges remain, including variable healing, risk of retear, and inconsistent clinical outcomes. A promising alternative is biological reinforcement using the iliotibial (IT) band, which may provide structural support and biological advantages while permitting a smaller graft harvest and tunnel size. This study evaluates IT band graft biomechanics in ACL repair using finite element analysis (FEA), focusing on graft thickness and tunnel-diameter compatibility.

**Methods:**

FE models simulated 10 mm wide IT band grafts of three thicknesses (1, 1.5, 2 mm) in femoral and tibial tunnels of 5, 6, and 7 mm. Knee anatomy was derived from CT data and processed in Blender, Gmsh, and FEBio. Bones were modeled as rigid, cartilage as hyperelastic Mooney–Rivlin, menisci as orthotropic Fung, and ligaments/grafts as fiber-reinforced hyperelastic materials. Contact included tied and sliding elastic formulations. The tibia was fully constrained in all six degrees of freedom, while femoral rotation was prescribed to achieve 30° of knee flexion. This position was maintained as a 100 N posterior femoral force was progressively applied. Von Mises stress and effective Lagrange strain distributions were analyzed to assess graft-tunnel compatibility.

**Results:**

Stress and strain increased when thicker grafts were paired with smaller tunnels. The 2 mm graft in a 5 mm tunnel showed peak stress of 11.62 MPa and strain of 2.5%. However, the 1.5-mm graft–6-mm tunnel configuration exhibited a lower maximum effective Lagrange strain of approximately 1.1%, a maximum principal stress of 5.69 MPa, and AP laxity of 3.5 mm, which was close to the intact-knee value of 3.2 mm. Larger tunnels consistently reduced stress and strain for all graft sizes. Peak stress and strain were localized near the femoral tunnel, identifying the region where graft stress and strain were highest.

**Discussion:**

FE analysis shows graft-tunnel mismatch compromises ACL repair mechanics. The 1.5-mm IT band graft with a 6-mm tunnel showed comparatively low graft stress and strain while maintaining AP laxity close to the intact-knee value. Tunnel diameter strongly influenced graft stress and strain, with smaller tunnels generally producing higher values.

**Limitation:**

A single patient-specific FE model was used, which may not capture anatomical variability. These findings provide computational support for further evaluation of the IT band as a biological internal brace and may inform future graft-selection and tunnel-sizing strategies in ACL repair.

## 1 Introduction

The anterior cruciate ligament (ACL) plays a crucial role in connecting the femur and tibia, thereby ensuring stability in the knee joint (Webster, 2021). ACL injuries have become more prevalent with the rising global popularity of sports, making up 30% of all knee injuries (Tang et al., 2021). ACL has limited intrinsic healing capacity following rupture because of its relatively low cellularity and vascularity (Pashaki et al., 2024). ACL reconstruction using a tendon graft remains the standard operative treatment for many active patients with complete ACL rupture (Murray et al., 2020). The surgical treatment of ACL ruptures has made significant progress over the years, but still has suboptimal outcomes, including prolonged return to sport and work, substantial graft harvest morbidity, persistent proprioceptive deficits (Fleming et al., 2022), elevated re-rupture rates in young high-demand athletes, and the progression of osteoarthritis (Ciceklidag et al., 2024; Murray and Fleming, 2013). These limitations, together with the desire to preserve the native ligament and its proprioceptive function, have renewed interest in primary ACL repair rather than complete ligament reconstruction. The appeal of primary ACL repair as opposed to reconstruction has led to the development of varied surgical techniques (Bachmaier et al., 2020), many of which have shown promising results in proximal tear patterns (Heusdens et al., 2019). However, in younger, higher-demand patients (Pashaki et al., 2024) and midsubstance tears, these techniques have not shown reproducible results (Vermeijden et al., 2021).

This limited intrinsic healing response led to the development of biologically augmented ACL-repair strategies designed to provide a provisional scaffold within the defect, as synovial fluid can interfere with formation and maintenance of the fibrin clot required for ligament healing (Murray and Fleming, 2013). Among the most extensively studied biologically augmented approaches is Bridge-Enhanced ACL Repair (BEAR), which uses a collagen-based scaffold to support healing of the native ACL (Proffen et al., 2015). Bioenhanced ACL repair has demonstrated favorable biomechanical outcomes in preclinical animal models, while a prospective randomized clinical trial involving complete midsubstance ACL tears demonstrated noninferior patient-reported outcomes and anteroposterior knee laxity for BEAR compared with autograft ACL reconstruction at 2 years (Murray et al., 2020). Clinical outcomes of ACL repair augmented with the InternalBrace (Arthrex, Inc., Naples, FL, USA) have been promising, demonstrating favorable patient-reported results and restoration of knee stability, particularly in cases of acute and proximal ACL tears (Bastos et al., 2025; Wilson et al., 2022). Currently available ACL repair and augmentation approaches continue to face limitations, including retear, the need for subsequent surgery, variable healing, and dependence on the mechanical integrity of the repair construct during the early healing period (Nwachukwu et al., 2019).

One potential area for improvement in ACL repair scaffold constructs could be the use of a Biological Internal Brace instead of a synthetic suture (Pardiwala and Lee, 2023). The IT band is particularly attractive for this purpose because it is an autologous, mechanically robust tissue that is already used clinically as a reinforcing structure in lateral extra-articular tenodesis procedures associated with ACL reconstruction. IT band procedures include harvesting a narrow strip while preserving its distal attachment at Gerdy’s tubercle, demonstrating the surgical feasibility of obtaining a relatively small autologous reinforcement graft (Abusleme et al., 2021). By serving as both a biological and structural support, and not the primary graft source, it may allow for a much smaller graft harvest (and corresponding tunnel size) to satisfy the time-zero stability requirements adequately. While the IT band is a robust structure, its relatively thin profile compared to the hamstring and patellar tendons can result in a smaller graft and tunnel diameter, which may limit its ability to provide time-zero stability for the healing ACL repair (Chan et al., 2010; Snaebjörnsson et al., 2017). In IT band-based ACL procedures, a narrow strip of the IT band is harvested from the lateral thigh and can be routed toward the knee while maintaining its distal attachment at Gerdy’s tubercle. The graft is passed deep to the lateral collateral ligament and directed toward the ACL femoral footprint. In ACL reconstruction, the femoral tunnel can be drilled using an outside-in guide toward the native femoral ACL footprint, while the tibial tunnel is created using an ACL guide directed toward the native tibial footprint (Gomes et al., 2017). In a recent study, a biomimetic scaffold was developed as part of an integrated ACL-repair construct in which a hollow scaffold was positioned around an internal IT band graft, combining mechanical reinforcement from the biological internal brace with scaffold-mediated support for tissue healing. The surgical feasibility of this concept was demonstrated through implantation of the IT band-based construct in human cadaver knees (Pashaki et al., 2026a; Pashaki et al., 2026b).

An important translational consideration for this approach is the dimensional compatibility between the IT band graft and the bone tunnel. Graft size and tunnel diameter are clinically related variables rather than independent geometric parameters. In ACL surgery, the prepared graft is typically assessed using a sizing block or sizing cylinder, and the corresponding bone tunnel is subsequently created using a guide pin and calibrated surgical reamers selected according to the graft dimensions (Pastrone et al., 2011). For an IT band construct, the graft width can be defined during surgical harvest; for example, IT band-based extra-articular procedures describe marking and harvesting a 10 mm wide strip (Abusleme et al., 2021). In contrast, IT band thickness is largely patient-specific. Sonographic anatomical studies have reported mean IT band thicknesses of approximately 1.1 ± 0.2 mm at the lateral femoral epicondyle and approximately 1.95 ± 0.30 mm at the femoral condyle, demonstrating clinically relevant variation in this dimension (Goh et al., 2003; Gyaran et al., 2011). Graft–tunnel dimensional matching is therefore important because graft thickness is primarily determined by patient anatomy, whereas tunnel diameter can be controlled surgically. Graft–tunnel diameter disparity is a recognized technical consideration in ACL surgery and has been specifically investigated in relation to graft–tunnel healing and mechanical fixation (Yamazaki et al., 2002).

Finite element analysis (FEA) provides an appropriate approach for investigating this question because it allows graft thickness and tunnel diameter to be varied systematically while maintaining controlled anatomy, material properties, fixation conditions, and external loading. In addition, FEA permits evaluation of local graft stress and strain distributions and joint kinematics that are difficult to measure directly throughout the graft *in vivo*. Previous ACL finite element studies have demonstrated the ability of this approach to identify regional graft stress concentrations and to evaluate the biomechanical consequences of changing tunnel diameter (Ren et al., 2021; Wang et al., 2020b). In the present study, these FE-derived stress and strain outputs are used to compare different IT band graft-thickness/tunnel-diameter combinations and determine how graft–tunnel dimensional compatibility influences the magnitude and spatial distribution of stress on the graft. These comparisons are intended to identify graft–tunnel relationships under the modeled loading condition and thereby provide a basis for future tunnel-sizing strategies. To our knowledge, no prior study has systematically investigated the interaction between IT band graft thickness and bone-tunnel diameter when the IT band is used as an intra-articular biological reinforcement for ACL repair.

Therefore, this study aimed to determine how graft thickness and bone-tunnel diameter influence the response of a 10 mm wide autologous IT band reinforcement construct during ACL repair. The 10 mm graft width was selected based on our previously demonstrated cadaveric implementation and clinically described IT band-based procedures, as discussed above. To do this, we systematically investigated the relationship between tunnel diameter and IT band graft thickness using FEA. IT band thicknesses of 1, 1.5, and 2 mm were evaluated in combination with 5, 6, and 7 mm bone tunnels to represent anatomically and surgically relevant variations in graft–tunnel geometry. The simulations were performed under a Lachman-type loading condition at 30° of knee flexion with a 100 N posterior femoral force, representing an early postoperative assessment. The analysis evaluated von Mises stress, maximum principal stress, effective Lagrange strain, spatial stress distribution, and anteroposterior joint displacement to characterize both graft-level mechanical loading and joint-level restraint. These outputs were compared across configurations to determine how graft–tunnel dimensional compatibility affects the behavior of the construct. The objective was not to establish a universally optimal surgical dimension, but rather to identify graft–tunnel relationships under the modeled loading condition that may inform future graft-sizing and tunnel-selection strategies.

## 2 Materials and methods

### 2.1 Study design: 3D modeling of the IT band embedded in the knee

Figure 1 outlines the workflow developed to simulate ACL repair surgery using the IT band and analyze its performance through FEA. The anatomical knee joint model used in this study was obtained from the Open Knee(s) project (Erdemir, 2016). The model was imported into Blender (Blender.org, 2026), where the ACL was removed, and the femoral and tibial tunnels were created through the NURBS curve using the Blender software. For IT band modeling, according to the literature, healthy volunteer studies have reported mean IT band thicknesses of approximately 1.1 ± 0.2 mm at the lateral femoral epicondyle and 1.95 ± 0.30 mm at the femoral condyle, whereas cadaveric measurements indicate that harvested IT band tissue may be less than 1 mm thick (Goh et al., 2003; Gyaran et al., 2011). Accordingly, 1 and 2 mm were selected as representative lower and upper values, respectively, while 1.5 mm was included as an intermediate value.

**FIGURE 1:**
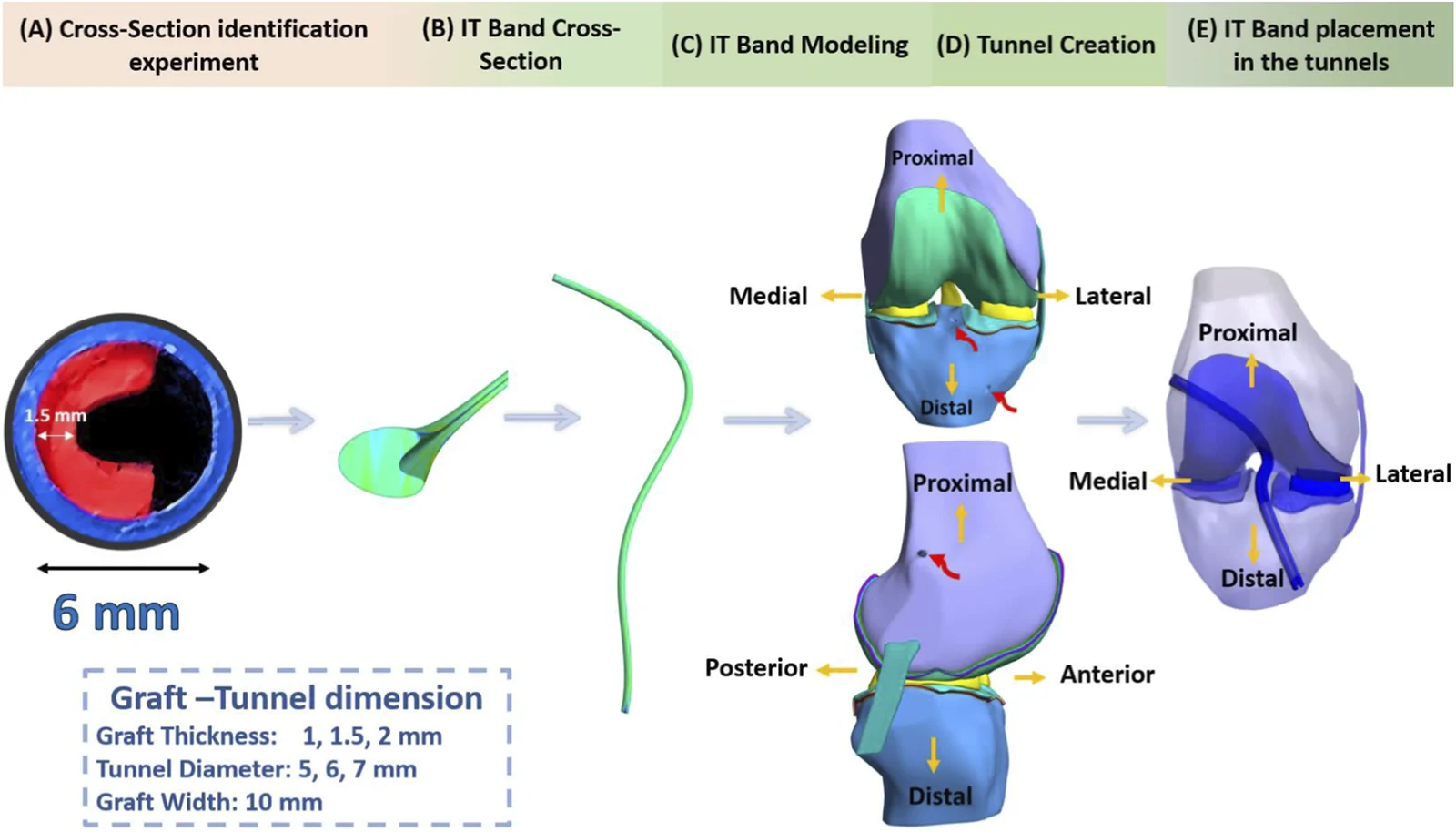
Workflow for finite element modeling of ACL repair using the iliotibial (IT) band graft. **(A)** The process begins with the identification of the IT band cross-section and tunnel configuration. **(B)** The cross-section is then modeled and reconstructed to preserve anatomical accuracy. **(C)** The IT band graft is modeled along the native ACL trajectory using Blender. **(D)** Femoral and tibial bone tunnels are created to simulate surgical drilling paths. **(E)** The IT band graft is inserted into the tunnels to complete the reconstructed knee model.

IT band-based lateral extra-articular tenodesis and modified Lemaire procedures have described tunnels or sockets ranging from approximately 5–7 mm (Eggeling et al., 2021; Haus et al., 2023; Puzzitiello et al., 2019). In one recent study, cadaveric implementation of the ACL-repair construct, a 10 mm wide IT band strip was passed through a 5–6 mm bone tunnel (Pashaki et al., 2026a). Therefore, tunnel diameters of 5, 6, and 7 mm were selected based on surgical precedent and the dimensions required to accommodate an IT band strip while minimizing bone removal.

In this study, the modeled IT band graft with a fixed width of 10 mm and thicknesses of 1, 1.5, and 2 mm passed through circular femoral and tibial tunnels with diameters of 5, 6, and 7 mm. The graft was modeled along the native ACL trajectory between the fixation regions; therefore, graft length was determined by this anatomical pathway and was not varied independently. The baseline femoral and tibial tunnel paths were directed along the native ACL trajectory toward the corresponding anatomical footprints. The graft was initialized in a standardized configuration within the tunnels to permit controlled comparison among configurations. Potential folding, buckling, twisting, or asymmetric packing of the compliant IT band strip during surgical passage was not explicitly modeled and is therefore considered an idealization of the graft–tunnel geometry.

Following the graft placement, the entire geometry, including the knee joint and the IT band graft, was exported and processed in Gmsh to convert the surface mesh into a high-quality volume mesh suitable for finite element modeling. The meshed models were then imported into the FEBio software (Maas et al., 2012), where material properties and physiological boundary conditions were applied. Simulations were performed to evaluate stress and strain distributions across the graft for different combinations of tunnel diameters (5 mm, 6 mm, and 7 mm) and IT band thicknesses (1 mm, 1.5 mm, and 2 mm) in combination with a 10 mm wide graft harvest.

## 3 Finite element model development

In this study, FEA was conducted to evaluate graft stress and strain under 30° knee flexion with a 100 N posterior femoral load. The simulations were performed using FEBio, an open-source finite element solver designed explicitly for modeling the large deformation biomechanics of soft tissues. The knee structures and IT band graft were assigned material properties based on experimental data reported in the literature. FEBio was used to evaluate graft stress and strain distributions and to identify regions of higher localized stress and strain.

### 3.1 Material modeling

All material properties used in the simulation were derived from values reported in the literature. The deformable soft-tissue structures, including the IT band graft, were discretized using tetrahedral volume meshes, whereas the femur and tibia were represented as rigid triangular surface meshes. To represent anatomical detail, the femur and tibia were modeled as single rigid bodies without separate cortical and trabecular regions, as the present analysis focused on graft-level mechanics rather than bone deformation or stress. To represent the articular soft-tissue anatomy, the femoral and tibial cartilages were segmented into three distinct layers, each characterized using an uncoupled hyperelastic Mooney–Rivlin material model to capture the nonlinear response of nearly incompressible soft tissues (Brown et al., 2009; Haut Donahue et al., 2002; Sophia Fox et al., 2009). The menisci were represented using an orthotropic Fung elasticity model (Yao et al., 2005), which accommodates their direction-dependent mechanical properties. Detailed material parameters for each anatomical structure are provided in Table 1.

**TABLE 1:** Principal constitutive models and material parameters used in the finite element model.

Structure	Constitutive model	Principal material parameters
Bone	Rigid body	No elastic constants assigned (Bae and Cho, 2020; Erdemir, 2013; Risvas et al., 2022)
Femoral and tibial cartilage	Mooney–Rivlin	K = 8 MPa, C1 = 0.856, C2 = 0 MPa (Brown et al., 2009; Erdemir and Sibole, 2010; Risvas et al., 2022)
Menisci	Orthotropic Fung	K = 10 MPa; E_1_ = 125 MPa; E_2_ = 27.5 MPa; E_3_ = 27.5 MPa; G_12_ = 2 MPa; G_23_ = 12.5 MPa; G_31_ = 2 MPa; υ_12_ = 0.10; υ_23_ = 0.33; υ_31_ = 0.10; c = 1 (Erdemir and Sibole, 2010; Risvas et al., 2022)
MCL and LCL	Transversely isotropic Mooney–Rivlin	K = 397 MPa; C_1_ = 1.44 MPa; C_2_ = 0 MPa; C_3_ = 0.57 MPa; C_4_ = 48; C_5_ = 467.1 MPa; Lam_max = 1.063 (Sibole and Erdemir, 2012; Weiss et al., 1996)
PCL	Transversely isotropic Mooney–Rivlin	K = 122 MPa; C_1_ = 3.25 MPa; C_2_ = 0 MPa; C_3_ = 0.1196 MPa; C_4_ = 87.178; C_5_ = 431.063 MPa; Lam_max = 1.035 (Sibole and Erdemir, 2012; Weiss et al., 1996)
IT band graft	Transversely isotropic Mooney–Rivlin	K = 10,000 MPa; C_1_ = 2.75 MPa; C_2_ = 0 MPa; C_3_ = 0.065 MPa; C_4_ = 112.0971; C_5_ = 490.668 MPa; Lam_max = 1.0182; fibers aligned with the longitudinal graft direction (Risvas et al., 2024; Weiss et al., 1996)

Ligaments were modeled as nearly incompressible, transversely isotropic, hyperelastic fiber-reinforced materials, incorporating a Mooney-Rivlin formulation for the ground matrix (Erdemir and Sibole, 2010; Maas et al., 2012). The mechanical behavior was described using the strain energy function given in Equations 1, 2.

W=C1 I1 −3+C2 I2−3+K2LnJ2+Fλ (1)

With

$$\lambda =\left\{\begin{array}{c} \begin{array}{c} 0\;\lambda <1\\ C_{3}\left(e^{C_{4}\left(\lambda -1\right)}-1\right)\;1\leq \lambda <\lambda_{m} \end{array}\\ {\;C}_{5}+C_{6}\lambda \;\lambda \geq \lambda_{m}\; \end{array}\right.$$ (2)

where λ is the stretch along the local fiber direction, and λm​ is the transition stretch. The principal constitutive parameters assigned to the cartilage, menisci, ligaments, and IT band graft are summarized in Table 1. The IT band graft was modeled as a nearly incompressible, transversely isotropic, fiber-reinforced hyperelastic material. The isotropic ground matrix was represented using a Mooney–Rivlin formulation, while the reinforcing collagen fibers were described using a nonlinear tension-only constitutive relationship. The material fiber direction was assigned locally along the longitudinal axis of the graft. The uncoupled strain-energy density function was expressed as shown in Equation 3:

$$\Psi =C_{1}\left(I_{1}-3\right)+C_{2}\left(I_{2}-3\right)+F_{f}\left(\lambda \right)+K/2{\left[\ln\left(J\right)\right]}^{2}$$ (3)

where I_1_ and I_2_ are the modified first and second invariants of the right Cauchy–Green deformation tensor, J is the determinant of the deformation gradient, K is the bulk modulus, and λ is the deviatoric stretch along the local fiber direction (Hammer et al., 2012; Risvas et al., 2024; Steinke et al., 2012; Weiss et al., 1996).

### 3.2 Contact modeling

The IT band graft was tied at localized terminal femoral and tibial fixation regions, representing idealized secure time-zero fixation, and was not bonded along the tunnel walls (Figures 2A,B). The remaining graft–bone interfaces, including the tunnel surfaces, were modeled using sliding-elastic contact, allowing contact and relative sliding while preventing penetration. The femur and tibia were represented as single rigid bodies; therefore, separate interactions with cortical and trabecular bone were not modeled. The rigid-bone and frictionless-contact assumptions do not distinguish cortical from trabecular graft contact and may affect local load transfer and sliding at the tunnel apertures; friction, abrasion, and fretting were not evaluated. An evaluation of graft–tunnel contact comparing frictionless contact (μ = 0) with μ = 0.3 showed only minor changes in peak stresses (≤3.2%), indicating that the results were minimally affected by the graft–tunnel friction condition.

**FIGURE 2:**
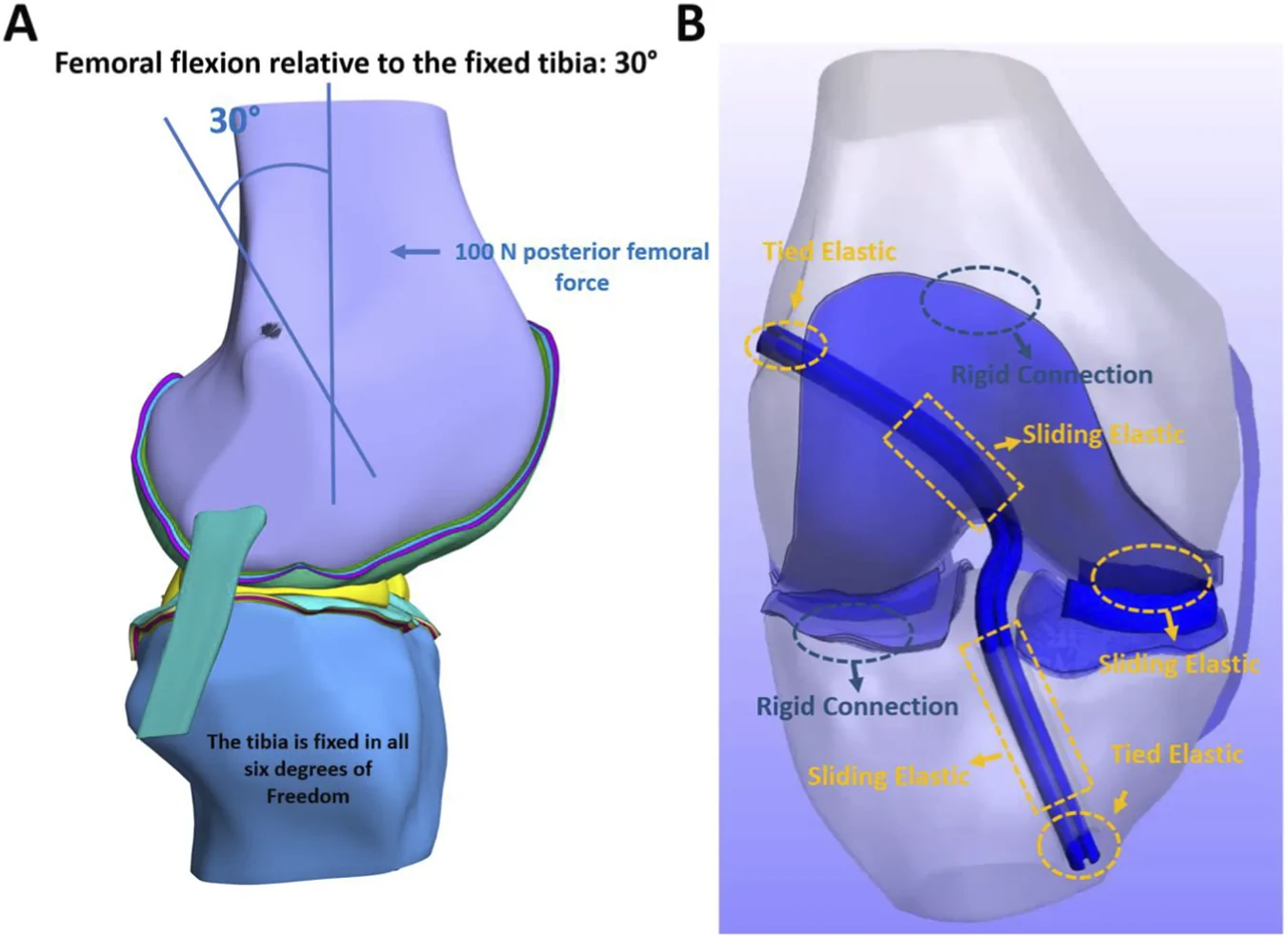
**(A)** Loading and boundary conditions: the tibia was fixed in all six degrees of freedom, the femur was positioned at 30° flexion, and a 100 N posterior femoral force was applied while maintaining the prescribed flexion angle. **(B)** Rigid, tied-elastic, and sliding-elastic contact definitions.

### 3.3 Loading and boundary conditions

To simulate an early-stage postoperative condition, the tibia was constrained in all six degrees of freedom and used as the fixed reference body. To establish a standardized Lachman-type test posture, a 30° femoral flexion rotation was prescribed about the model-defined mediolateral flexion axis. Once the 30° posture was reached, the flexion angle was maintained while a 100 N posterior femoral force was progressively applied. The femur was not restricted to pure anterior–posterior; the remaining unconstrained degrees of freedom were allowed to respond to the applied load and joint interactions. With the tibia fixed, posterior femoral translation represents the relative anterior–posterior joint motion analogous to anterior tibial translation during the Lachman examination. Risvas et al. (2022) simulated the Lachman maneuver using a fixed tibia, 30° of knee flexion, and a 134 N posterior femoral force, while Wang et al. (2020a) applied a 103 N anterior tibial load in an ACL reconstruction model. The selected 100 N force magnitude is also comparable to the 103 N anterior tibial load used in previous ACL reconstruction FE studies. The loading and boundary conditions are illustrated in Figures 2A,B. AP laxity was also quantified as the posterior displacement of the femoral reference point relative to the fixed tibia at the end of the 100 N loading step.

### 3.4 Mesh convergence analysis

A mesh convergence analysis was performed to assess the mesh independence of the FE predictions. The 1.5 mm graft combined with a 6 mm tunnel was used as the representative configuration, and the graft mesh was progressively refined from 7,174 to 55,970 elements while maintaining identical material properties, loading conditions, boundary conditions, and contact definitions. The maximum von Mises stress and maximum effective Lagrange strain within the graft were extracted for each mesh refinement level, and the percentage change between successive refinements was evaluated. As shown in Figure 3A, both graft stress and strain progressively approached stable values with increasing mesh density, with only minor changes observed between the two finest mesh levels. Accordingly, a target element size of 0.9 mm, corresponding to approximately 30,000 elements throughout the IT-band graft, was selected for the subsequent analyses, as further refinement to 55,970 elements produced only minor changes in maximum von Mises stress and maximum effective Lagrange strain.

**FIGURE 3:**
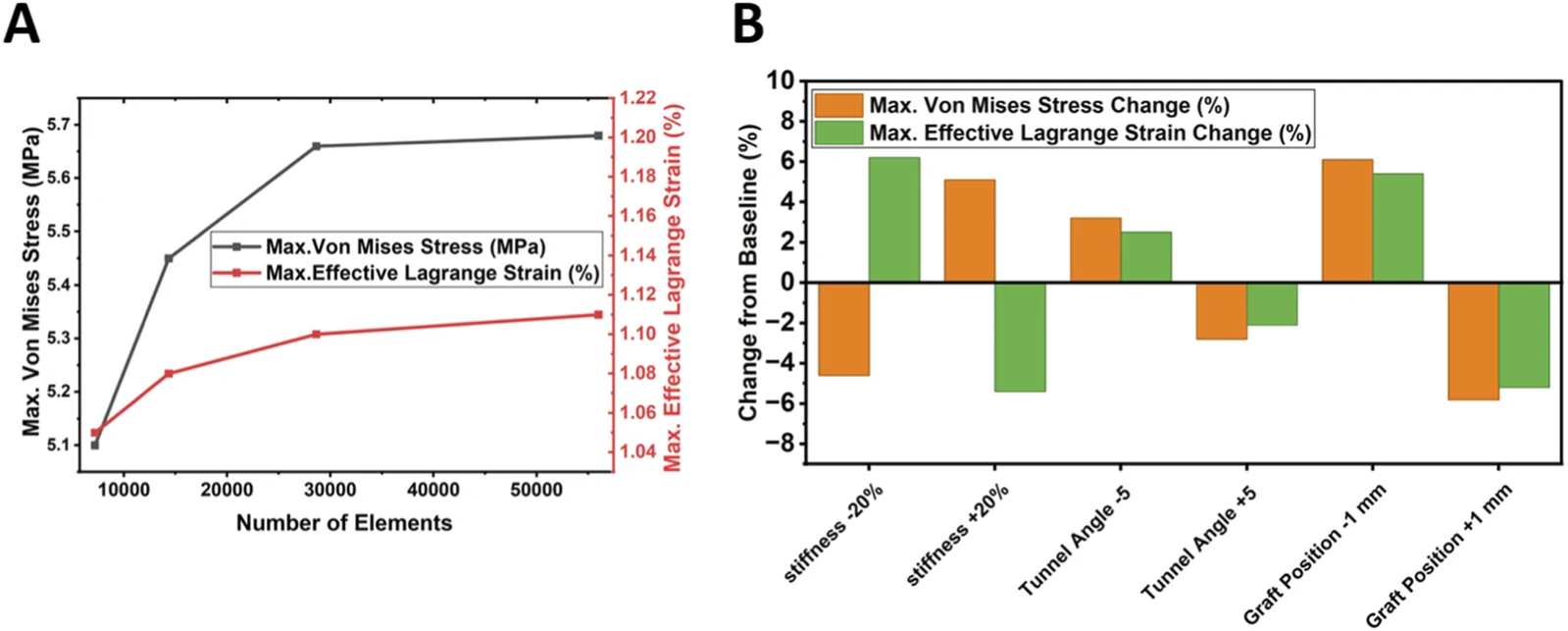
Mesh convergence analysis for the 1.5-mm graft–6-mm tunnel configuration **(A)** Sensitivity analysis of graft stiffness (±20%), tunnel orientation (±5°), and graft position (±1 mm) on maximum von Mises stress and maximum effective Lagrange strain **(B)**.

### 3.5 Sensitivity analysis

For sensitivity analysis, a 1.5 mm graft combined with a 6 mm tunnel was used as the baseline configuration. Three parameters were independently varied relative to their baseline values: graft stiffness by ±20%, tunnel orientation by ±5°, and graft position by ±1 mm. For each sensitivity case, all other model parameters, including geometry, loading conditions, boundary conditions, contact definitions, and mesh settings, were maintained unchanged. The maximum von Mises stress and maximum effective Lagrange strain within the graft were extracted for each simulation. The sensitivity of stress and strain was quantified as the percentage change relative to the corresponding baseline value. The analysis showed that the investigated parameter variations produced relatively limited changes in the predicted results, generally within approximately 2%–6%, with graft position producing the greatest overall influence and tunnel orientation the smallest. These variations were selected as moderate sensitivity ranges rather than defined surgical-error thresholds. They produced approximately 2%–6% changes in graft stress and strain, compared with approximately 26%–40% changes in peak von Mises stress associated with changes in tunnel diameter. The results of the sensitivity analysis are presented in Figure 3B. An additional sensitivity analysis applied 2% initial reference strain to the representative 1.5-mm graft/6-mm tunnel configuration to evaluate its effect on AP laxity and maximum principal graft stress.

## 4 Results

The distribution of von Mises stress within the graft under varying conditions of graft thickness (1 mm, 1.5 mm, and 2 mm) and tunnel diameter (5 mm, 6 mm, and 7 mm) is presented in Figure 4. A clear relationship emerged between graft-tunnel dimensions and stress concentration, indicating that both parameters critically influence the graft. For the thinnest graft (1 mm), the highest stress was observed when the tunnel diameter was smallest (5 mm), reaching approximately 9.52 MPa. As the tunnel diameter increased to 6 mm and 7 mm, the stress values decreased to 6.05 MPa and 4.21 MPa, respectively (Figures 4A1–A3). A similar trend was observed for the 1.5 mm graft, where stress decreased from 9.07 MPa at a 5 mm tunnel diameter to 5.66 MPa and 4.10 MPa at 6 mm and 7 mm tunnel diameters, respectively. For the thickest graft (2 mm), the 5-mm tunnel produced the highest overall stress (11.62 MPa) (Figures 4C1–C3). Normalization using the tunnel-to-graft cross-sectional area ratio showed that lower ratios generally corresponded to higher von Mises stress. However, similar normalized ratios did not always produce equivalent stresses, indicating that graft stress depends on graft–tunnel dimensional compatibility rather than on a specific graft thickness. Maximum principal stress showed a similar tunnel-dependent trend to von Mises stress, generally decreasing with increasing tunnel diameter for each graft thickness. The 2.0-mm graft with a 5-mm tunnel showed the highest von Mises and maximum principal stresses (11.62 and 12.5 MPa, respectively), whereas the 1.5-mm graft with a 6-mm tunnel showed substantially lower values (5.66 and 5.69 MPa, respectively). These results indicate that the differences observed using von Mises stress were also reflected in the tensile-oriented maximum principal stress (Table 2).

**FIGURE 4:**
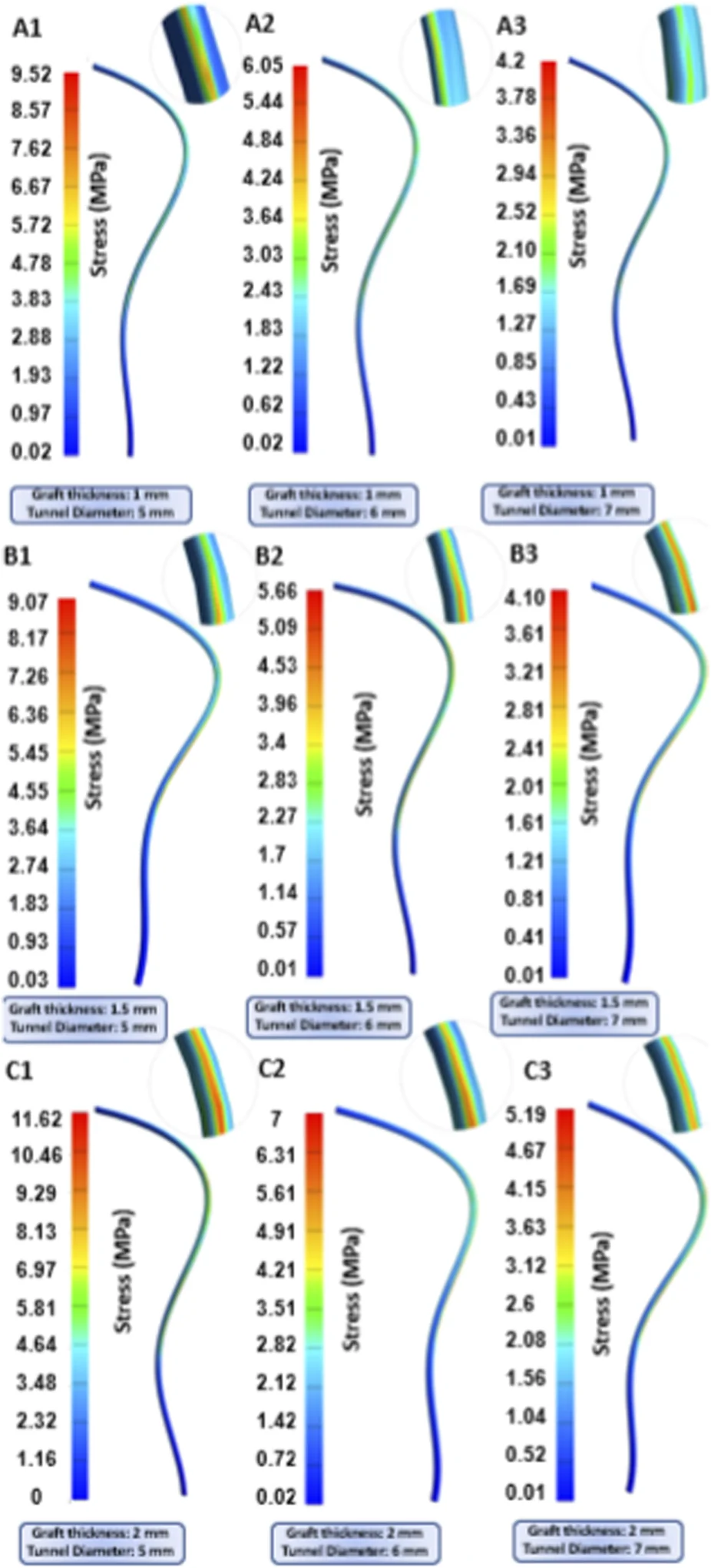
Von Mises stress distribution in IT Band grafts with different graft thicknesses and tunnel diameters. The 1 mm graft with tunnel diameters of 5 mm, 6 mm, and 7 mm, respectively **(A1–A3)**. The 1.5 mm graft with tunnel diameters of 5 mm, 6 mm, and 7 mm **(B1–B3)**. The 2 mm graft with tunnel diameters of 5 mm, 6 mm, and 7 mm **(C1–C3)**.

**TABLE 2:** Maximum principal stress, Von Mises stress, anterior–posterior laxity, ACL and PCL force sharing across the evaluated graft–tunnel configurations.

Graft thickness	Tunnel diameter	Peak von Mises stress (MPa)	Maximum principal stress (MPa)	AP laxity (mm)	ACL force (N)	PCL force (N)	ACL/PCL ratio
Intact knee	—	—	—	3.2	110.0	3.0	36.7
1 mm	5 mm	9.52	6.6	4.8	108.0	3.3	32.7
1 mm	6 mm	6.05	5.5	5.1	107.0	3.5	30.6
1 mm	7 mm	4.21	4.5	5.5	106.0	3.8	27.9
1.5 mm	5 mm	9.07	7.7	3.9	107.0	3.1	34.5
1.5 mm	6 mm	5.66	5.69	3.5	109.0	3.0	36.3
1.5 mm	7 mm	4.10	5.3	4.1	108.0	3.2	33.8
2 mm	5 mm	11.62	12.5	3.3	105.0	3.0	35.0
2 mm	6 mm	7.01	9.3	3.4	106.0	3.0	35.3
2 mm	7 mm	5.19	7.5	3.7	107.0	3.1	34.5

The corresponding effective Lagrange strain results in Figure 5 provide complementary insights into the performance of each tunnel graft configuration. As shown in Figure 5A, effective strain generally decreases with increasing tunnel diameter, while increasing IT band thickness results in elevated strain values across all tunnel sizes. For instance, with a 1 mm thick IT band graft, the maximum strain decreases from approximately 1.5% in the 5 mm tunnel to around 0.4% in the 7 mm tunnel. This pattern suggests that larger tunnels reduce geometric constraint and local strain concentration within the graft. Conversely, thicker grafts experienced higher effective Lagrange strain under the evaluated tunnel constraints. Figures 5B1,B2 show representative strain distributions for the 2-mm graft–5-mm tunnel and 1.5-mm graft–6-mm tunnel configurations, respectively. The 2-mm graft–5-mm tunnel configuration exhibited the highest maximum effective Lagrange strain of approximately 2.5%, whereas the 1.5-mm graft–6-mm tunnel configuration exhibited a lower value of approximately 1.1%.

**FIGURE 5:**
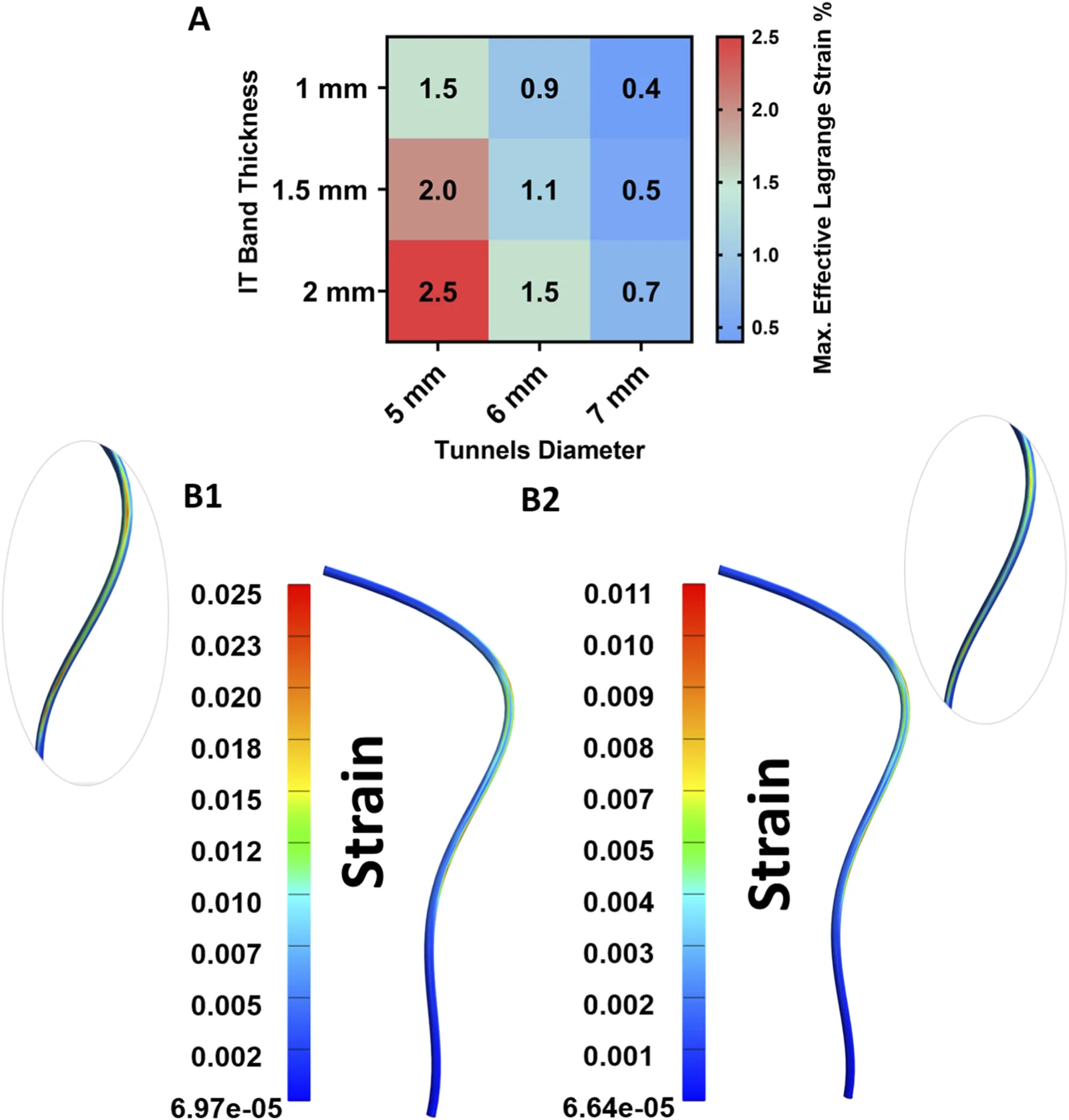
Effective Lagrange strain across IT band graft thicknesses and tunnel diameters. **(A)** Heat map of maximum effective Lagrange strain. **(B1)** Effective Lagrange strain distribution for the 2-mm graft–5-mm tunnel configuration (maximum = 2.5%). **(B2)** Effective Lagrange strain distribution for the 1.5-mm graft–6-mm tunnel configuration (maximum = 1.1%).

At 30° of knee flexion under the 100 N posterior femoral load, the intact-knee model demonstrated an AP laxity of 3.2 mm. AP laxity for all configurations ranged from 3.3 to 5.5 mm across the graft–tunnel configurations, compared with 3.2 mm for the intact knee. At the end of the same loading step, the ACL force was approximately 110 N, whereas the PCL force was approximately 3 N, confirming that the ACL was the predominant cruciate restraint during AP translation (Table 2). Across the configurations, ACL forces ranged from 105 to 109 N and PCL forces from 3.0 to 3.8 N, with ACL/PCL ratios of 27.9–36.3 compared with 36.7 in the intact knee. The predicted ACL force was comparable to the experimental value of 114.1 ± 7.4 N reported at 30° under a 100 N anterior shear load, while the minimal PCL loading was consistent with its limited contribution to restraint of anterior tibial translation (Butler et al., 1980; Xerogeanes et al., 1995). The 1.5-mm graft–6-mm tunnel configuration showed 3.5 mm of AP laxity, whereas the 1.0-mm graft–7-mm tunnel configuration exhibited the greatest laxity (5.5 mm), indicating that lower graft stress did not necessarily correspond to greater joint restraint. Despite differences in AP laxity across the graft–tunnel configurations, ACL force at the 100 N loading endpoint remained within a relatively narrow range of 105–109 N, whereas peak graft stresses varied substantially across configurations. This indicates that the observed differences in local graft stress were not simply proportional to changes in joint laxity or ACL force and were also strongly influenced by graft–tunnel geometry. The friction evaluation of the most constrained 2.0-mm graft/5-mm tunnel configuration produced ≤3.2% changes in peak stress, while introducing 2% initial reference strain in the representative 1.5-mm graft/6-mm tunnel configuration reduced AP laxity from 3.50 to approximately 3.30 mm and modestly increased maximum principal graft stress from 5.69 to approximately 6.0 MPa.

Although IT band thickness is subject to patient-specific anatomical variability and cannot be precisely controlled, our findings emphasize the importance of avoiding tunnel overstuffing, which may occur when a relatively thick graft is inserted into a disproportionately narrow tunnel. In the present model, relatively thick grafts paired with smaller tunnels were associated with higher graft stress and strain. These observations highlight the interdependent effects of tunnel diameter and graft thickness on the immediate response of the IT band graft.

## 5 Discussion

### 5.1 Comparison with previous computational and biomechanical findings

The finite element results present a comprehensive evaluation of how tunnel diameter and IT band graft thickness influence stress and strain distributions in IT band–augmented ACL repair. Hammer et al., (2012) reported a mean ultimate tensile stress of 35.8 ± 16.4 MPa. In another study, Zwirner et al. (2019) reported a mean ultimate tensile stress of approximately 29 ± 17 MPa for human IT band specimens. Seeber et al. (2020) reported deformation of 7.0% ± 1.9% at the yield point and 11.3% ± 4.2% at ultimate failure, whereas the maximum effective Lagrange strain predicted in the present study was approximately 2.5%. Nevertheless, these comparisons should not be interpreted as direct failure criteria because the experimental measurements were obtained under longitudinal uniaxial tension, whereas the present model reports maximum von Mises stress and effective Lagrange strain under a multiaxial loading state.

The present models predicted maximum graft von Mises stresses ranging from approximately 4.1–11.62 MPa across the investigated graft thicknesses and tunnel diameters. These values are of a similar order of magnitude to those reported in previous ACL reconstruction finite element studies, although direct numerical comparison is limited by differences in graft geometry, material constitutive models, fixation methods, loading conditions, and boundary conditions. Wang et al. (2020c) reported a maximum ACL graft stress of approximately 15.4 MPa in a cadaveric-knee finite element model subjected to combined anterior tibial, internal rotational, and valgus loading, with stress concentration occurring at the graft redirection region near the tunnel entrance. In addition, Santos et al. (2023) reported a peak ACL stress of approximately 11 MPa in a finite element investigation under a high-demand combined loading condition, which is comparable to the upper range predicted in the present study. The maximum effective Lagrange strain predicted across the investigated graft configurations ranged from approximately 0.4%–2.5%. These values are comparable in magnitude to the relatively low ACL strains reported during controlled rehabilitation activities. Fleming et al. (1998) measured peak *in vivo* ACL strains ranging from approximately 1.2%–2.1% during stationary bicycling, with a mean peak strain of 1.7%. Similarly, Heijne et al. (2004) reported ACL strains of approximately 1.8%–2.0% during forward lunges and approximately 2.5%–2.6% during step-up and step-down exercises. Fleming et al. (1999) also measured mean peak ACL strains of approximately 2.69% and 2.76% during stair climbing at two different cadences, values that are close to the upper range predicted in the present study. Nevertheless, direct numerical comparisons should be interpreted with appropriate caution because the experimental studies quantified local strain in the native ACL, whereas the present study evaluated effective strain in an IT band graft under different constitutive assumptions, reference configurations, fixation strategies, and loading conditions.

AP laxity was also quantified from posterior femoral displacement under the applied 100 N load. Because the tibia was fixed, posterior femoral translation represents relative AP displacement and is mechanically analogous to anterior tibial translation during a Lachman-type test (Risvas et al., 2022). The intact-knee model exhibited 3.2 mm of AP laxity, while the graft–tunnel configurations ranged from 3.3 to 5.5 mm. The 1.5-mm graft–6-mm tunnel configuration showed 3.5 mm of AP laxity, close to the intact-knee response. For comparison, Kim et al. (2021) reported 2.89 mm of simulated anterior tibial translation under a 100 N load, compared with 2.43 mm experimentally. These values support the kinematic plausibility of the AP displacement, although differences in geometry, boundary conditions, and loading prevent direct numerical equivalence. ACL force at the 100 N endpoint remained similar across the graft–tunnel configurations despite substantial differences in peak graft stress. This suggests that the observed variation in local graft stress cannot be attributed to differences in AP laxity alone and is consistent with an important contribution from graft–tunnel geometry. During passive flexion, the model exhibited approximately 4.0° of femoral external rotation, 1.5° of valgus rotation, and a 0.8 N·m flexion reaction moment at 30°. This response is consistent with low-load passive knee behavior reported experimentally (Wilson et al., 2000; Sommers et al., 2004). Because external loading can alter tibiofemoral kinematics (Wünschel et al., 2012), these values are interpreted as physiologically plausible rather than as direct numerical validation.

### 5.2 Spatial distribution of graft stress and model limitations

Graft stress was not uniformly distributed along its length. Higher stresses were concentrated mainly near the femoral and tibial tunnel transition regions, consistent with the regional stress analysis in Figure 6A. These regions correspond to changes in graft direction between the intraosseous and intra-articular portions, resulting in localized tensile loading and bending together with graft–tunnel contact. Figure 6A further shows that, for the 2-mm graft, the 5-mm tunnel produced the highest regional stresses, with the femoral region exceeding 10 MPa and the tibial region exhibiting higher stress than the midsection. Increasing the tunnel diameter to 7 mm reduced the stress across the femoral, midsection, and tibial regions. Maximum principal stress showed a similar tunnel-dependent trend across the evaluated configurations (Table 2). Maximum effective Lagrange strain decreased with increasing tunnel diameter for all evaluated graft thicknesses (Figure 6B). These stress peaks identify regions of higher graft loading but should not be interpreted as predicted failure sites because damage and rupture were not modeled. A limitation is that peak von Mises and maximum principal stresses were extracted independently and may not occur at the same graft location. Their relative magnitudes therefore should not be used alone to infer a specific local stress state, and neither metric can be considered definitively superior for the present anisotropic graft model. The graft-harvesting and tunnel-placement procedure and the corresponding graft stress and strain results are summarized in Figure 7.

**FIGURE 6:**
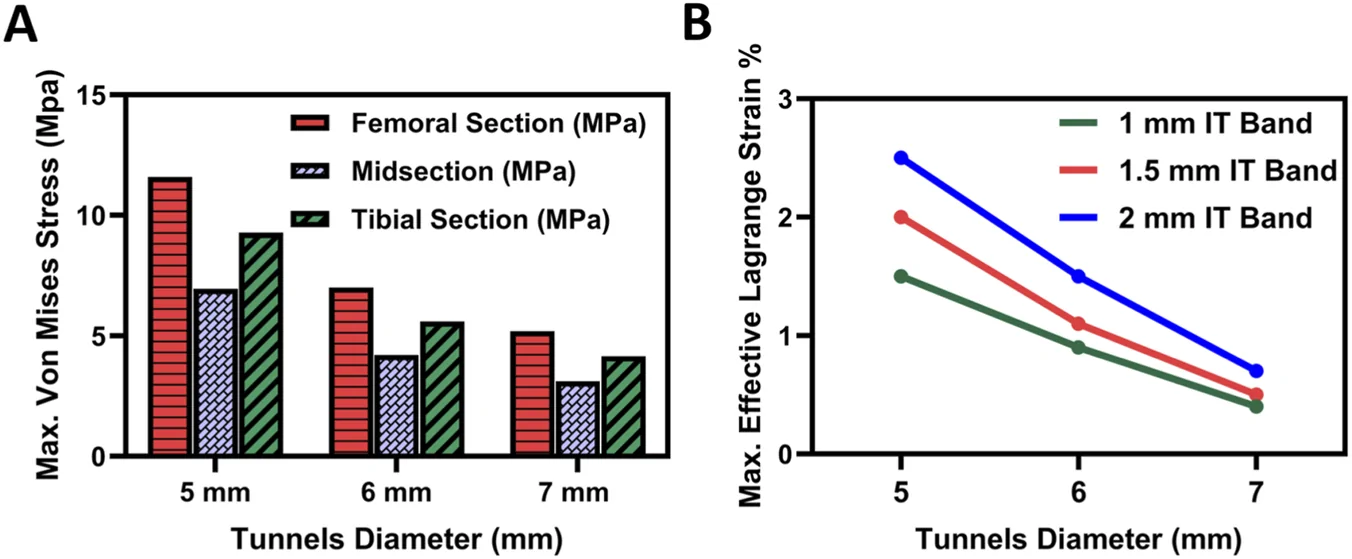
**(A)** Maximum von Mises stress across the femoral, midsection, and tibial regions of the 2-mm graft for tunnel diameters of 5, 6, and 7 mm. **(B)** Maximum effective Lagrange strain (%) as a function of tunnel diameter for IT band graft thicknesses of 1, 1.5, and 2 mm.

**FIGURE 7:**
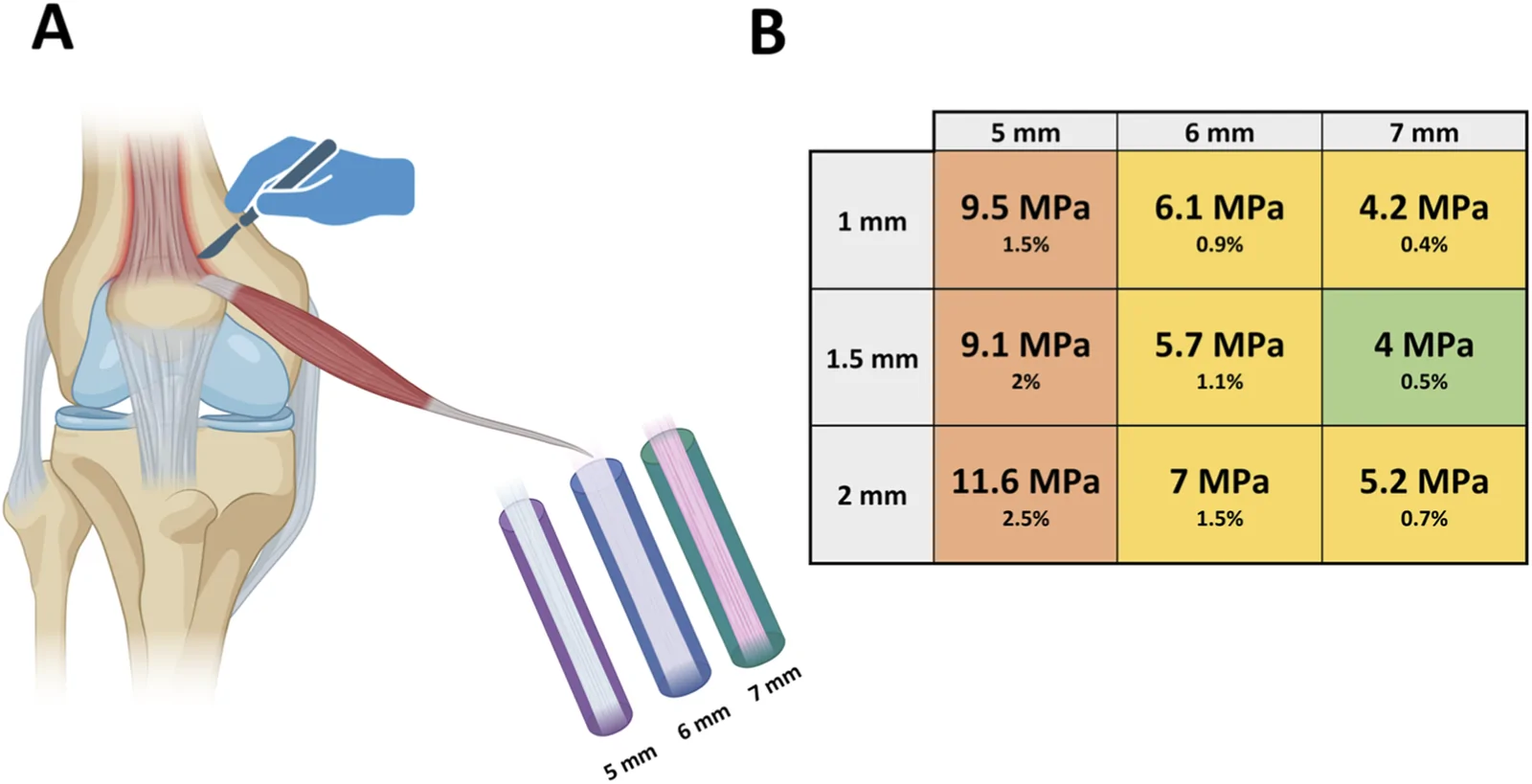
Schematic illustration of IT band graft harvesting and placement into bone tunnels of varying diameters (5 mm, 6 mm, and 7 mm) for IT-ACL repair surgery **(A)**. Table summarizing von Mises stress (MPa) and corresponding maximum effective Lagrange strain (%) in IT band grafts with varying thicknesses (1 mm, 1.5 mm, 2 mm) across different tunnel diameters (5 mm, 6 mm, 7 mm) **(B)**. Color coding: green = comparatively low stress/strain; yellow = intermediate stress/strain; orange = comparatively high stress/strain within the evaluated configurations. The color categories are study-specific and do not correspond to established clinical failure thresholds. These study-specific categories are intended solely for comparative visualization and are not based on established physiological, safety, yield, or failure thresholds. Figure 7A has been created with biorender.com.

Because native IT band thickness varies among patients, whereas tunnel diameter can be selected during surgery, tunnel sizing should be matched to the harvested graft dimension to avoid graft overstuffing. However, because graft–tunnel contact pressure, micromotion, fixation loss, tissue damage, and cyclic effects were not directly evaluated, these potential consequences should be regarded as biomechanical interpretations rather than demonstrated clinical outcomes.

A key limitation is that the model was based on a single CT-derived knee geometry and therefore does not capture inter-subject anatomical variability. The 1.5-mm graft–6-mm tunnel result should therefore be interpreted as specific to the modeled geometry rather than universally optimal. In addition, muscle forces and dynamic tibiofemoral loading were not included, so the findings are limited to comparative trends under the prescribed low-load condition.

Another limitation is the lack of direct experimental validation. Although the surgical feasibility of the IT band construct was demonstrated in a previous cadaveric study (Pashaki et al., 2026b), graft stress, strain, and joint displacement were not measured under comparable loading conditions. Therefore, the present results should be interpreted primarily as comparative trends, and future validation should include direct graft-strain and joint-kinematic measurements.

No initial surgical pretension was applied. Because pretension and fixation angle can influence graft force, joint constraint, and stress–strain behavior, this assumption may affect the predicted results (Bae and Cho, 2020; Brady et al., 2007; Gertel et al., 1993; Peña et al., 2005). The zero-pretension condition may affect predicted stress, strain, and joint constraint, although it was applied consistently across all configurations to isolate graft-thickness and tunnel-diameter effects. Similarly, because the friction and initial-condition sensitivity analyses were performed on selected configurations, their exact effects may vary across graft–tunnel combinations; however, the observed changes were modest in the tested cases. In addition, the rate-independent hyperelastic model does not capture viscoelasticity, strain-rate effects, cyclic fatigue, damage, rupture, or long-term remodeling.

## 6 Conclusion

This study systematically evaluated the effects of IT band graft thickness and tunnel diameter on graft stress, strain, and AP laxity using finite element analysis. Increasing tunnel diameter generally reduced von Mises stress, maximum principal stress, and effective Lagrange strain; however, lower graft stress did not necessarily correspond to greater joint restraint. The 1.0-mm graft–7-mm tunnel configuration produced low graft stress and strain but the greatest AP laxity (5.5 mm), whereas the 2.0-mm graft–5-mm tunnel configuration produced AP laxity close to the intact knee (3.3 vs. 3.2 mm) but the highest graft stress and strain. In contrast, the 1.5-mm graft–6-mm tunnel configuration exhibited a lower maximum effective Lagrange strain of approximately 1.1% and AP laxity of 3.5 mm.

These findings indicate that graft–tunnel compatibility should be evaluated using both graft loading and joint restraint rather than graft stress alone. Within the specific anatomy, loading condition, and modeling assumptions evaluated, the 1.5-mm graft–6-mm tunnel configuration showed a favorable combination of these outcomes. Further studies incorporating multiple subject-specific anatomies, experimental validation, graft pretension, additional loading conditions, and long-term biological effects are needed to determine whether these relationships are maintained under more physiologic conditions.

## Data Availability

The raw data supporting the conclusions of this article will be made available by the authors, without undue reservation.
